# Electrolyte imbalance markers and acid–base disturbances in Brazilian male runners during a 45-km mountain ultramarathon

**DOI:** 10.7717/peerj.21308

**Published:** 2026-05-27

**Authors:** Marcelo Romanovitch Ribas, Danieli Isabel Romanovitch Ribas, Georgian Badicu, Samuel Ferreira, Marco Aurelio Telles Matta, Elto Legnani, Luca Paolo Ardigò, Julio Cesar Bassan

**Affiliations:** 1 Universidade Tecnologica Federal do Paraná, Curtiba, Brazil; 2 Human Genetics Laboratory, Centro Universitário Autônomo do Brasil (UniBrasil), Curtiba, Brazil; 3 Department of Physical Education and Special Motricity, Faculty of Physical Education and Mountain Sports, Transilvania University of Braşov, Braşov, Romania; 4 Department of Teacher Education, NLA University College, Oslo, Norway

**Keywords:** Ultramarathon, Acid–base equilibrium, Muscle fatigue, Potassium, Endurance running

## Abstract

**Objective:**

This study investigated electrolyte and acid–base alterations and their associations with performance in 40 Brazilian male runners during a 45-km mountain ultramarathon.

**Method:**

The following parameters were assessed before and after the race: pH, sodium (Na^+^), potassium (K^+^), calcium (Ca^2+^), glucose, carbon dioxide pressure (pCO_2_), partial pressure of oxygen (pO_2_), bicarbonate (HCO_3_), hematocrit and lactate.

**Results:**

Potassium levels decreased significantly across all performance groups (Fastest: *p* = 0.004; Moderate-Fast: *p* = 0.004; Moderate-Slow: *p* = 0.003; Slowest: *p* = 0.004), while lactate increased in the Fastest (*p* = 0.03), Moderate-Fast (*p* = 0.002), and Slowest (*p* = 0.03) groups. Sodium increased in the Moderate-Fast group (*p* = 0.005), whereas calcium decreased in the Slowest group (*p* = 0.005). Both pCO_2_ and HCO_3_ were significantly reduced across all groups (pCO_2_: *p* = 0.0003–0.0005; HCO_3_: *p* = 0.003–0.0004), and pH decreased in the Slowest group (*p* = 0.01). Between-group differences were observed between Fastest and Slowest for pH (*p* = 0.02), pCO_2_ (p = 0.001), and K^+^ (*p* < 0.001), and between Fastest and Moderate-Slow for Na^+^ (*p* = 0.03). Negative correlations were found between race time and post-race pH (*R* = −0.46), Ca^2+^ (*R* = −0.33), and Na^+^ (*R* = −0.31). In the Slowest group, Δ K^2+^ was strongly correlated with race time (*R* = −0.83; *p* = 0.003).

**Conclusion:**

Performance in a 45-km mountain ultramarathon appears to be closely associated with the ability to preserve acid–base and electrolyte homeostasis, particularly regarding potassium, sodium, calcium and pH.

## Introduction

Endurance competitions that extend beyond the traditional marathon distance expose athletes to exceptional physical and metabolic stress. Among these events, ultramarathons demand sustained neuromuscular efficiency, optimized cardiovascular regulation, and precise control of acid–base and electrolyte homeostasis throughout hours of continuous effort (Knechtle & Nikolaidis, 2018; Scheer et al., 2022). Ultramarathon running has grown substantially in recent decades, with increasing global participation among recreational and elite athletes (Scheer, 2019). This expansion has intensified scientific interest due to the pronounced physiological stress associated with prolonged endurance exercise and its implications for performance optimization and athlete safety (Knechtle & Nikolaidis, 2018; Scheer et al., 2022).

Mountain ultramarathons represent a particularly demanding research model within ultra-endurance running, as prolonged exercise duration is combined with challenging terrain and environmental conditions, leading to marked variability in physiological and performance responses (Eichenberger et al., 2012). A 45-km mountain ultramarathon exceeds the traditional marathon distance while remaining accessible to a broad population of trained runners, making it a relevant real-world model for investigating physiological disturbances without the logistical constraints of longer ultra-distance events (Knechtle & Nikolaidis, 2018).

During such prolonged exertion, progressive alterations in sodium (Na^+^), potassium (K^+^), calcium (Ca^2^^+^), and bicarbonate (HCO_3_^−^) levels may compromise muscular contraction and energy metabolism, thereby impairing performance and recovery capacity (Costa et al., 2019; Ribas et al., 2025). Tiller et al. (2022) highlighted that these biochemical responses are highly dependent on multiple interacting variables, including hydration dynamics, ventilatory control, and environmental stress, which can influence endurance performance and recovery. Similar observations have been reported by Costa et al. (2019), who demonstrated that dehydration, thermal stress, and ionic shifts during prolonged exercise can contribute to impaired muscular performance and an increased risk of fatigue in ultra-endurance athletes.

Field-based investigations in ultramarathon runners have revealed substantial interindividual variability in physiological and biochemical responses to prolonged endurance exercise, even among athletes with comparable training status and performance levels (Hoppel et al., 2019). This variability reflects the multifactorial nature of endurance performance, with electrolyte balance and acid–base regulation being influenced by race duration, terrain profile, hydration strategies, and individual tolerance to sustained metabolic stress (Costa et al., 2019; Tiller et al., 2022; Hoppel et al., 2019). However, evidence obtained under real mountain ultramarathon conditions remains limited, particularly when biochemical measurements are collected immediately before and after competition.

In a previous investigation involving Brazilian female ultramarathoners, Ribas et al. (2025) observed marked post-race reductions in bicarbonate (HCO_3_^−^) and carbon dioxide pressure (pCO_2_), suggesting a ventilatory adjustment to compensate for metabolic acidosis. Concurrently, declines in potassium (K^+^) and hematocrit (Hct) among slower and intermediate performers were interpreted as evidence of hemodilution and fluid imbalance associated with prolonged effort (Ribas et al., 2025). Despite the general stability of sodium (Na^+^), moderate performers exhibited a positive association between post-race Na^+^ levels and race duration, reinforcing the importance of sodium regulation in delaying fatigue during sustained exercise (Ribas et al., 2025). However, whether similar biochemical patterns occur in male mountain ultramarathon runners and how these responses relate to performance remains unclear.

Sex-related differences in physiological regulation such as greater lean mass, higher hemoglobin concentration, and distinct endocrine responses in men, may significantly influence the metabolic stress and ionic shifts that occur during ultra-endurance races (Scheer et al., 2022; Ribas et al., 2025; Campa et al., 2021). Therefore, investigating male runners in real-world mountain ultramarathon conditions may help clarify how electrolyte and acid–base disturbances manifest and how they associate with race performance.

Given the limited evidence concerning biochemical regulation in male ultramarathon runners, further investigation is essential to understand how electrolyte and acid–base disturbances manifest in this population. The present study therefore aimed to examine ionic and acid–base dynamics in Brazilian male athletes competing in a 45-km mountain ultramarathon and to determine their association with race time, thereby contributing to a broader understanding of the physiological mechanisms underpinning endurance performance. We hypothesized that slower runners would exhibit greater disturbances in electrolyte balance and acid–base regulation compared with faster runners.

## Materials and Methods

### Design

This cross-sectional field investigation was performed during the Ultramaratona Perdidos SkyMarathon^®^, a 45-km mountain race located in Tijucas do Sul, Paraná, Brazil. The course involved an accumulated ascent of approximately 2,630 m, exposing athletes to considerable altitude and terrain variability. The event started at 6:00 a.m., under temperatures of about 13 °C that gradually increased to 19 °C, with relative humidity oscillating between 70% and 90%. Climatic parameters, including ambient temperature and humidity, were obtained from local meteorological monitoring, given their potential impact on thermoregulation and electrolyte behavior during endurance competition.

### Participants

Sample size estimation was conducted in G*Power 3.1.9.2, adopting a repeated-measures ANOVA (within–between interaction) with *α* = 0.05, statistical power = 0.80, and two time points (pre- and post-race). A large effect size (*f* = 0.40), consistent with Hoppel et al. (2021) and Kishi et al. (2024), guided the calculation, indicating that at least 16 participants would ensure sufficient sensitivity. The final cohort comprised 40 amateur male runners (mean age 38.3 ± 6.9 years), which increased the effective power (∼0.84) and strengthened the detection of physiological differences related to race performance.

Eligibility required athletes to possess 3–4 years of mountain-running experience, prior completion of at least two races exceeding 21 km and one over 42 km, and consistent training (5–6 sessions per week, 1–2 h daily and >3 h on weekends). Weekly training volume and intensity were verified through a structured self-report questionnaire. Exclusion criteria included lack of informed consent, incomplete data collection, withdrawal, or conditions affecting fluid regulation (metabolic disorders, medication use, or musculoskeletal injury). After screening 149 registrants, 40 qualified for participation.

Runners were divided into four quartile-based performance groups according to finishing times. This quartile-based categorization was adopted to allow stratified comparisons across distinct performance levels, representing a methodological approach widely used in sports science. Previous studies, such as Dausin et al. (2026), have applied quartile-based approaches to stratify continuous variables and compare physiological responses across groups.

Group 1 (Fastest Times): Times ranged from 312.18 to 446.6 min (*n* = 10).

Group 2 (Moderate-Fast Times): Times ranged from 447.2 to 480.9 min (*n* = 10).

Group 3 (Moderate-Slow Times): Times ranged from 484.1 to 558.6 min (*n* = 10).

Group 4 (Slowest Times): Times ranged from 595.6 to 668.7 min (*n* = 10).

Ethical approval was granted by the Research Ethics Committee of Faculdade Dom Bosco, Paraná, Brazil (protocol 2.275, 040 14 September 2017), in accordance with Resolution 466/12 of the Brazilian National Health Council.

### Instruments and procedures

Data collection was organized into two experimental stages: the pre-race stage, conducted during the official kit distribution on the day before the competition, and the post-race stage, performed immediately after completion of the 45-km mountain ultramarathon.

At both stages, capillary blood samples were obtained to assess biochemical and acid–base markers. The following variables were analyzed: hydrogen ion concentration (pH), sodium (Na^+^), potassium (K^+^), calcium (Ca^2^^+^), glucose (Glu), partial pressure of carbon dioxide (pCO_2_), partial pressure of oxygen (pO_2_), blood lactate (Lac), hematocrit (Hct), and bicarbonate (HCO_3_^−^). These parameters were selected because they provide a comprehensive overview of electrolyte regulation, acid–base equilibrium, and metabolic stress during ultra-endurance exercise. Blood collection and handling were performed by a trained nurse, following sterile procedures to ensure consistency and minimize the risk of contamination. Post-race blood samples were collected within 2–3 min after race completion, following a standardized protocol to minimize the influence of early recovery-related physiological fluctuations.

### Anthropometric evaluation

Anthropometric assessment included measurements of total body mass (TBM, kg), height, body mass index (BMI, kg/m^2^), body fat (BF%, kg), lean mass (LM, %, kg), and total body water (TBW, %, L). TBM was measured using a platform scale (Filizola^®^, São Paulo, Brazil; 0.1 kg precision), and height with a stadiometer (Seca^®^, Hamburg, Germany; 0.1 cm precision), considering the mean of three measurements. Body composition was evaluated using whole-body tetrapolar bioelectrical impedance analysis (Maltron^®^, Rayleigh, UK; 50 kHz), according to the manufacturer’s equation and standardized procedures (Velázquez-Alva et al., 2022). Anthropometric measurements were performed before and after the race; however, post-race bioelectrical impedance data were not analyzed due to the inability to ensure standardized measurement conditions under field settings and were therefore used only for sample characterization.

### Blood sampling and biochemical analysis

Capillary blood samples were collected *via* fingertip puncture using heparinized 200 µL capillaries (Roche^®^ Capillary 250). The puncture site was sterilized with 70% alcohol prior to collection, and samples were analyzed immediately after withdrawal. Biochemical analyses were conducted using a GEM Premier 3000 gas analyzer (Instrumentation Laboratory^®^, Bedford, MA, USA), which is validated for rapid clinical and sports-science applications (Waskiewicz et al., 2012; American Board of Internal Medicine, 2022).

Parameters measured included hydrogen ion concentration (pH), sodium (Na^+^), potassium (K^+^), calcium (Ca^2^^+^), glucose (Glu), partial pressure of carbon dioxide (pCO), partial pressure of oxygen (pO), blood lactate (Lac), hematocrit (Hct), and bicarbonate (HCO^−^), all considered key indicators of metabolic stress and ionic regulation during endurance performance. Equipment calibration followed manufacturer recommendations before and during the field assessment. The analyzer provided results within approximately 85 s, ensuring real-time data collection immediately after race completion. Previous research has confirmed strong correlations (*r* = 0.91–0.99) between this analyzer and laboratory-based reference systems, validating its use under field conditions (Bénéteau-Burnat et al., 2004).

### Statistical analysis

All analyses were performed in R 4.0.5. Data normality was verified by the Shapiro–Wilk test. To assess intra- and inter-group differences, repeated-measures ANOVA (ANOVA-RM) with Greenhouse–Geisser correction was applied when assumptions were violated. Partial eta-squared (*η*^2^*p*) was used to quantify effect sizes. Between-group comparisons were conducted *via* one-way ANOVA followed by Tukey’s *post-hoc* test (*p* ≤ 0.05). Statistical power (%) was calculated to confirm reliability. Pearson correlation analyses were performed treating race time as a continuous variable, reporting *r*, *R*^2^, and *p*-values. Stratified analyses by performance quartile explored group-specific associations. Graphical visualizations were produced with ggplot2 and ggpubr packages.

## Results

Table 1 presents the body composition variables of Brazilian male ultramarathon runners, categorized by performance level prior to the 45 km race. Significant between-group differences were observed for total body mass (TBM; *p* = 0.03), body mass index (BMI; *p* = 0.02), body fat mass (BF, kg; *p* = 0.03), lean mass (LM kg; *p* = 0.05), and total body water (TBW, L; *p* = 0.02). *Post-hoc* analysis indicated that these differences occurred primarily between the Fastest and Moderate-Fast groups. Effect sizes were large (*η*^2^*p* > 0.14) for these variables, with statistical power exceeding 70%. No significant differences were found for body fat percentage (BF%), lean mass percentage (LM%), or TBW percentage (TBW%) (*p* > 0.05).

**Table 1:** Comparison of pre-race body composition between performance groups in ultramarathon runners.

Variable	Fastest (Mean ± SD)	Moderate-fast (Mean ± SD)	Moderate-slow (Mean ± SD)	Slowest (Mean ± SD)	*p-value*	Partial Eta^2^	Effect size f	Achieved power (%)
TBM (Kg)	70.5 ± 7.3	80.5 ± 9.8	78.5 ± 9.0	72.6 ± 5.9	0.03^*^	0.22	0.533	76.9
BMI	23.0 ± 1.3	26.1 ± 2.9	25.5 ± 1.5	24.4 ± 2.6	0.02^*^	0.23	0.556	80.7
BF (%)	9.8 ± 1.4	11.5 ± 2.2	11.6 ± 2.0	10.7 ± 2.7	0.22	0.11	0.357	40.6
BF (kg)	6.9 ± 1.2	9.4 ± 2.1	9.1 ± 1.7	7.9 ± 2.4	0.02^*^	0.23	0.549	79.5
LM (Kg)	63.7 ± 6.7	72.1 ± 8.7	69.5 ± 8.4	64.8 ± 4.7	0.05^*^	0.19	0.497	70.4
LM (%)	90.2 ± 1.4	88.5 ± 2.2	88.4 ± 2.0	89.3 ± 2.7	0.22	0.11	0.357	40.6
TBW (L)	44.6 ± 6.8	52.8 ± 6.4	50.9 ± 6.2	47.4 ± 3.4	0.02^*^	0.24	0.569	82.8
TBW(%)	66.1 ± 1.0	64.8 ± 1.6	64.7 ± 1.5	65.4 ± 2.0	0.23	0.11	0.354	39.9

**Notes.**

* *p* ≤ 0.05.

TBM total body mass (kg) BMI body mass index (kg/m^2^) BF (%) body fat percentage BF (kg) absolute fat mass (kg) LM (kg) lean mass (kg) LM (%) lean mass percentage TBW (L) total body water (L) TBW (%) total body water percentage

Table 2 summarizes electrolyte concentrations before and after the 45-km mountain ultramarathon across performance groups. Potassium (K^+^) concentrations decreased significantly from pre- to post-race in all groups (Fastest: *p* = 0.004; Moderate-Fast: *p* = 0.004; Moderate-Slow: *p* = 0.003; Slowest: *p* = 0.004). Sodium (Na^+^) concentrations increased significantly in the Moderate-Fast group (*p* = 0.005), while no significant changes were observed in the other groups (*p* > 0.05). Calcium (Ca^2^^+^) concentrations decreased significantly only in the Slowest group (*p* = 0.005). Blood lactate concentrations increased significantly in the Fastest (*p* = 0.03), Moderate-Fast (*p* = 0.002), and Slowest (*p* = 0.03) groups. No significant between-group differences were detected for the remaining electrolyte variables (*p* ≥ 0.05).

**Table 2:** Variations in electrolyte levels in Brazilian male ultramarathon runners before and after a 45 km mountain race.

Group	Variable	Pre (Mean ± SD)	Post (Mean ± SD)	*F*	*p-Value*	Partial Eta^2^	Achieved power (%)
Fastest (*n* = 10)	K^+^ (mmol/L)	5.4 ± 1.0	4.4 ± 0.5	14.8	0.004^*^	0.62	92.2
	Lac (mmol/L)	2.2 ± 0.7	3.2 ± 1.2	6.6	0.03^*^	0.42	65.6
Moderate-fast (*n* = 10)	Na^+^ (mmol/L)	142.1 ± 3.0	145.1 ± 1.9	14.0	0.005^*^	0.61	93.2
	K^+^ (mmol/L)	4.81 ± 0.4	4.2 ± 0.3	14.4	0.004^*^	0.62	94.1
	Lac (mmol/L)	1.9 ± 0.6	2.9 ± 0.8	18.8	0.002^*^	0.68	98.0
Moderate-slow (*n* = 10)	K^+^ (mmol/L)	5.0 ± 0.6	4.05 ± 0.4	17.14	0.003^*^	0.66	95.7
Slowest (*n* = 10)	K^+^ (mmol/L)	5.1 ± 0.5	4.2 ± 0.3	14.9	0.004^*^	0.62	90.1
	Ca^2+^ (mmol/L)	1.24 ± 0.07	1.16 ± 0.04	13.7	0.005^*^	0.60	93.2
	Lac (mmol/L)	1.9 ± 0.4	2.7 ± 0.7	6.6	0.03^*^	0.42	65.6

**Notes.**

* *p* ≤ 0.05.

K^+^ potassium Lac Lactate Na^+^ sodium Ca^2^^+^ calcium mmol/L millimole per liter

Table 3 presents acid–base variables before and after the race. Partial pressure of carbon dioxide (pCO) decreased significantly in all performance groups (*p* range: 0.0003–0.0005). Bicarbonate (HCO_3_^−^) concentrations also declined significantly across all groups (*p* range: 0.003–0.0004). A significant reduction in pH was observed only in the Slowest group (*p* = 0.01). No significant changes were found for partial pressure of oxygen (pO) or hematocrit (Hct) (*p* ≥ 0.05).

**Table 3:** Alterations in acid-base balance parameters before and after a 45 km ultramarathon.

Group	Variable	Pre (Mean ± SD)	Post (Mean ± SD)	F	*p-Value*	Partial Eta^2^	Achieved power (%)
Fastest (*n* = 10)	pCO_2_ (mmHg)	39.9 ± 2.2	33.8 ± 2.4	33.9	0.0003^*^	0.79	99.9
	HCO_3_^−^(mmol/L)	26.39 ± 1.9	22.7 ± 2.3	15.7	0.003^*^	0.64	95.7
Moderate-fast (*n* = 10)	pCO_2_ (mmHg)	40.8 ± 2.2	33.8 ± 2.6	27.6	0.001^*^	0.75	99.7
	HCO_3_^−^(mmol/L)	4.81 ± 0.4	4.2 ± 0.3	24.7	0.001^*^	0.73	99.5
Moderate-slow (*n* = 10)	pCO_2_ (mmHg)	41.1 ± 2.8	33.2 ± 3.3	41.0	0.0001^*^	0.82	99.9
	HCO_3_^−^(mmol/L)	26.5 ± 1.4	22.2 ± 2.6	21.8	0.001^*^	0.70	98.7
Slowest (*n* = 10)	pH	7.42 ± 0.01	7.39 ± 0.02	10.1	0.01^*^	0.53	83.6
	pCO_2_(mmHg)	40.8 ± 2.7	33.8 ± 3.0	28.3	0.0005^*^	0.76	99.8
	HCO_3_^−^(mmol/L)	26.2 ± 2.0	20.5 ± 2.5	29.9	0.0004^*^	0.77	99.9

**Notes.**

* *p* ≤ 0.05.

pCO_2_ partial pressure of carbon dioxide mmHg millimeters of mercury HCO_3_^−^ bicarbonate mmol/L millimole per liter pH hydrogen potential

Post-race comparisons between performance groups are shown in Table 4. Significant differences were observed between the Fastest and Slowest groups for pH (*p* = 0.02), pCO_2_ (*p* = 0.001), and potassium (K^+^; *p* < 0.001). Sodium (Na^+^) concentrations differed significantly between the Fastest and Moderate-Slow groups (*p* = 0.03). No other post-race variables differed significantly between groups (*p* ≥ 0.05).

**Table 4:** Comparison of blood parameters among different running performance groups before and after a 45 km ultramarathon.

Variable	Fastest (Mean ± SD)	Moderate-fast (Mean ± SD)	Moderate-slow (Mean ± SD)	Slowest (Mean ± SD)	Significant comparison	*p-Value*
pH	7.42 ± 0.02	7.41 ± 0.02	7.40 ± 0.03	7.39 ± 0.03	Fastest *vs.* Slowest	0.02^*^
pCO_2_ (mmHg)	41.2 ± 2.5	39.8 ± 2.8	38.5 ± 3.1	37.1 ± 3.4	Fastest *vs.* Slowest	0.001^*^
Na^+^ (mmol/L)	143.0 ± 1.5	144.2 ± 1.6	142.7 ± 1.7	141.8 ± 1.9	Fastest *vs.* Moderate-Slow	0.03^*^
K^+^ (mmol/L)	4.92 ± 0.3	4.68 ± 0.4	4.35 ± 0.4	4.12 ± 0.5	Fastest *vs.* Slowest	*p* < 0.001^*^

**Notes.**

* *p* ≤ 0.05.

pH hydrogen potential pCO_2_ partial pressure of carbon dioxide mmHg millimeters of mercury Na^+^ sodium K^+^ potassium mmol/L millimole per liter

Pearson correlation analyses treating race time as a continuous variable are presented in Table 5. Race time was negatively correlated with post-race pH (*r* = −0.46, *p* = 0.002), Ca^2^^+^ (*r* = −0.33, *p* = 0.04), and Na^+^ (*r* = −0.31, *p* = 0.05). In the Slowest group, the change in potassium concentration (ΔK^+^) was strongly correlated with race time (*r* = −0.83, *p* = 0.003). No significant correlations were observed between race time and the remaining biochemical or anthropometric variables (*p* > 0.05).

**Table 5:** Correlation between biochemical markers (post-race) and performance (race time).

Variable	(Mean ± SD)	*R*	*R* ^2^	*p-Value*
pH	7.42 ± 0.03	−0.462	0.214	0.002^*^
Ca^2+^ (mmol/L)	1.18 ± 0.04	−0.331	0.110	0.04^*^
Na^+^ (mmol/L)	143.78 ± 2.75	−0.313	0.098	0.05^*^

**Notes.**

* *p* ≤ 0.05.

pH hydrogen potential Ca^2^^+^ calcium Na^+^ sodium mmol/L millimole per liter

**Figure 1:**
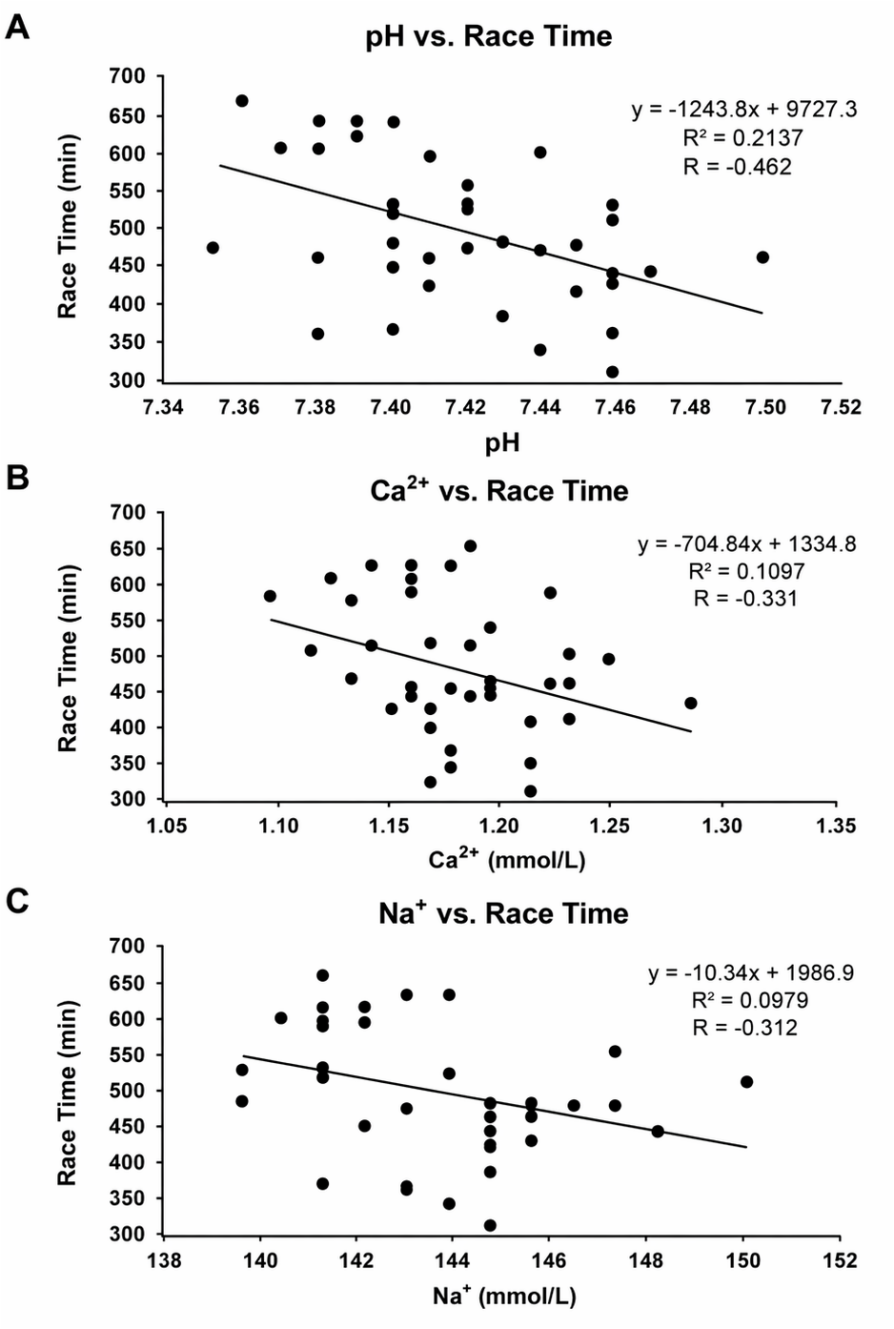
Relationships between post-race biochemical markers and race time (min) in Brazilian male ultramarathon runners during a 45-km mountain race. (A) Relationship between blood pH and race time. (B) Relationship between ionized calcium concentration (Ca^2+^, mmol/L) and race time. (C) Relationship between sodium concentration (Na^+^, mmol/L) and race time.A significant negative correlation was observed (R = −0.462; *R*^2^ = 0.2137; *p* = 0.002), indicating that higher systemic alkalinity was associated with faster race completion times. A moderate negative correlation was identified (R = −0.331; *R*^2^ = 0.1097; *p* = 0.04), suggesting that higher Ca^2+^ concentrations are associated with improved neuromuscular maintenance and shorter race times. A significant negative correlation was detected (R = −0.312; *R*^2^ = 0.0979; *p* = 0.05), indicating that higher sodium concentrations after the race were related to faster performance and better electrolyte regulation.

**Figure 2:**
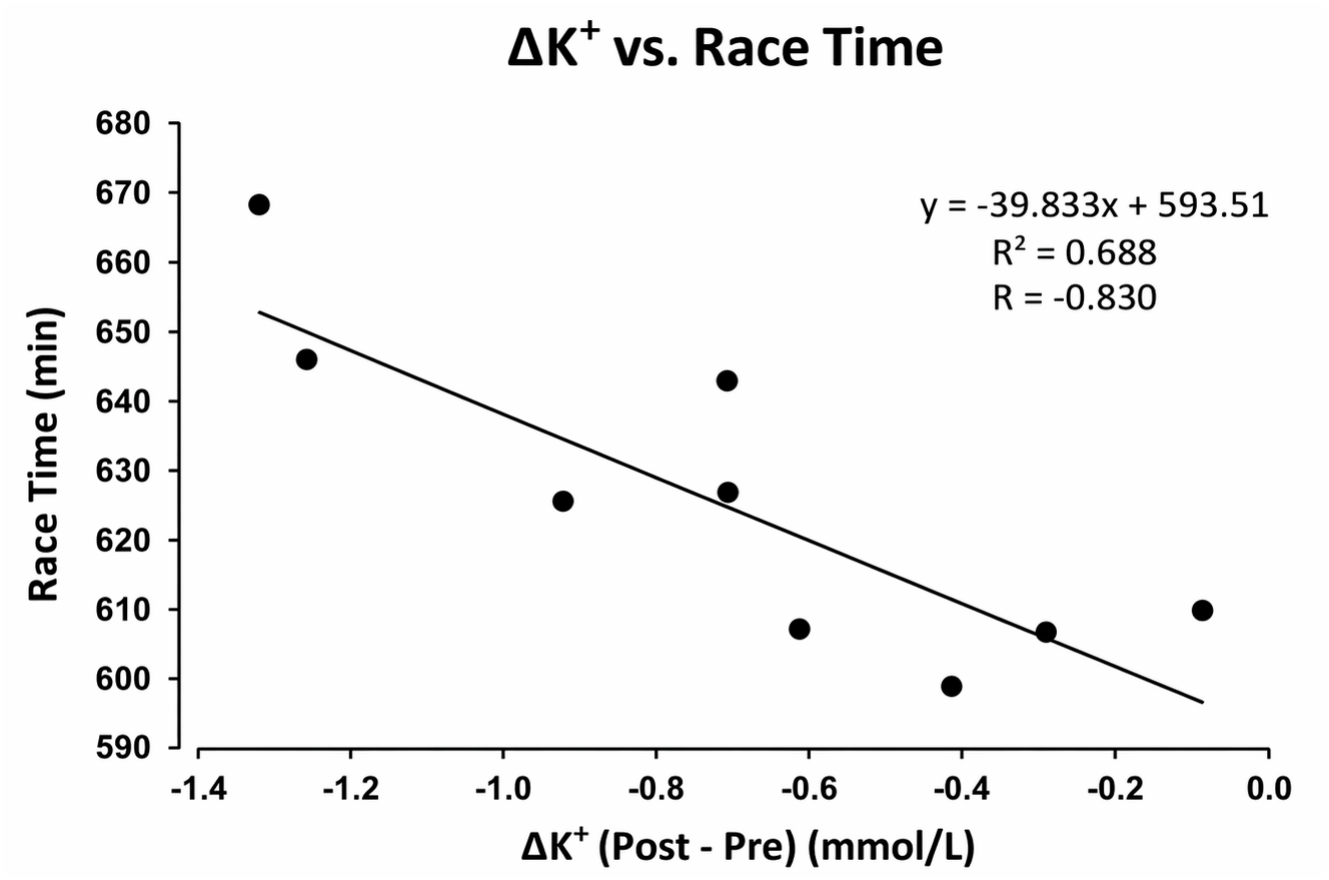
Relationship between potassium variation (ΔK^+^, post–pre) and race time (min) in the Slowest group. A strong negative correlation was observed (R = −0.83; *p* = 0.003), indicating that runners who exhibited greater reductions in K^+^ during the race tended to complete the ultramarathon in less time. This suggests that more pronounced potassium efflux may reflect higher neuromuscular activation and metabolic demand during prolonged exertion.

Figure 1 and 2 present graphical representations of the correlations described in Table 5 and are included solely for visual illustration. No additional results beyond those reported in the tables are presented.

## Discussion

The present study explored the dynamics of electrolyte and acid–base balance in Brazilian male ultramarathon runners during a 45-km mountain event, and how these parameters related to finishing time. Our hypothesis, that post-race alterations would occur in all participants and be more pronounced in slower runners, was partially supported. Higher post-race pH, Na^+^, and Ca^2^^+^ were associated with faster race times, suggesting that the ability to maintain metabolic homeostasis may be associated with improved endurance performance, rather than implying a direct causal relationship.

Regarding biochemical responses, all performance groups exhibited significant reductions in K^+^, consistent with ultra-endurance literature indicating that post-race potassium declines are commonly observed and have been associated with neuromuscular fatigue during prolonged exercise (Waskiewicz et al., 2012). The greater magnitude of K^+^ decline observed in slower runners may reflect increased susceptibility to ionic imbalance during prolonged exertion. The increase in Na^+^ observed in the intermediate group may be related to fluid and electrolyte regulation mechanisms described in endurance literature (Costa et al., 2019). Similarly, reductions in Ca^2^^+^ among slower finishers may reflect perturbations in muscle ionic balance during prolonged exercise, given calcium’s fundamental role in excitation–contraction coupling in skeletal muscle physiology (Pham & Puckett, 2023).

The post-race rise in lactate (Lac) indicates that anaerobic metabolism contributes intermittently during long-duration mountain races. These elevations likely reflect transient increases in exercise intensity across steep and technical terrain. Similar lactate responses have been documented in ultra-endurance competitions (Jastrzebski et al., 2015; Scheer et al., 2019). Accordingly, lactate concentrations should be interpreted as indicators of exercise intensity rather than solely as markers of fatigue (Subbarayalu et al., 2024). Comparable lactate increases were previously observed in Brazilian female ultramarathon runners competing in the same 45-km event (Eichenberger et al., 2012), supporting the reproducibility of this physiological response.

Reductions in pCO and HCO^−^ across all groups, coupled with decreased pH in slower athletes, point to a mild metabolic acidosis counterbalanced by ventilatory adjustments. Comparable trends were described in 100-km runners by Jastrzebski et al. (2015) and in 24-hour ultramarathoners by Waskiewicz et al. (2012). These findings are consistent with those seen in Brazilian female runners, who displayed similar declines in HCO_3_^−^ and pCO_2_ and lower pH among slower performers (Ribas et al., 2025).

Direct comparisons among performance groups showed that faster athletes presented higher post-race pH, Na^+^, and K^+^ values, indicating a more preserved electrolyte and acid–base profile. Similar patterns have been reported in studies comparing elite and amateur ultramarathon runners, where differences in biochemical stability across performance levels were observed (Tiller et al., 2022; Rüst et al., 2012). Tiller et al. (2022) highlighted that physiological differences between sexes may influence metabolic responses to prolonged exertion, reinforcing the relevance of stratified analyses in endurance research. In longer ultra-endurance events, such as 100-km races, amateur competitors frequently exhibit marked reductions in pH, HCO_3_^−^, and pCO_2_ accompanied by elevated lactate concentrations (Jastrzębski et al., 2015), a pattern comparable to the metabolic stress observed in slower runners in the present study. Overall, these findings suggest that the maintenance of internal biochemical balance during prolonged exercise is associated with better endurance performance.

Anthropometric variables also distinguished faster athletes, who exhibited lower total body mass, BMI, and body fat, along with higher lean mass and total body water. These characteristics are consistent with evidence indicating that training-induced morphological adaptations, such as reduced fat mass and greater lean mass, are associated with improved physiological efficiency and endurance performance in prolonged events (Tiller et al., 2022; Rüst et al., 2012).

Correlation analyses demonstrated negative associations between race time and post-race pH, Na^+^, and Ca^2^^+^. These correlations indicate that runners who maintained higher post-race values tended to finish faster, corroborating findings from female ultramarathoners in the same race (Ribas et al., 2025). The strong association between ΔK^+^ and race time among slower runners suggests that potassium variation may serve as a sensitive marker of physiological strain, consistent with observations from 24-hour ultramarathons (Waskiewicz et al., 2012). Collectively, these findings provide applied implications for ultra-endurance training and monitoring. Systematic assessment of electrolyte and acid–base status may assist in performance optimization and health risk reduction. Personalized hydration and electrolyte strategies may be preferable to uniform protocols, potentially reducing the risk of dehydration or hyponatremia, as discussed in endurance and hydration literature (Costa et al., 2019). Training approaches targeting lactate threshold development may also enhance tolerance to metabolic stress, although intervention-based studies are required to confirm these effects (Subbarayalu et al., 2024).

This study benefited from real-race data collection, immediate post-race analysis, and a robust sample size. Nevertheless, limitations should be acknowledged. Individual variability in hydration and nutritional strategies may have influenced biochemical responses, and the inclusion of male athletes only limits generalization to female populations. Future research should adopt longitudinal and interventional designs, integrating biochemical, nutritional, and recovery variables, to further elucidate mechanisms underpinning ultramarathon performance.

In addition, although anthropometric and bioelectrical impedance measurements were obtained before and after the race, post-race bioimpedance assessments could not be performed under fully standardized conditions inherent to field-based studies. Therefore, post-race bioimpedance data were not used for inferential analyses, which may limit the interpretation of hydration-related changes.

Future research should adopt longitudinal and interventional designs, integrating biochemical, nutritional, and recovery variables, to further elucidate mechanisms underpinning ultramarathon performance.

## Conclusion

This study highlights that both anthropometric characteristics and physiological regulation of electrolyte and acid–base balance are decisive for endurance performance in male ultramarathon runners. Athletes with lower body mass, higher lean tissue, and greater total body water were able to sustain metabolic stability more effectively throughout the 45-km mountain race. Faster runners exhibited better preservation of pH, Na^+^, and Ca^2^^+^, whereas slower athletes showed marked reductions in these parameters together with a more pronounced decline in K^+^. The consistent post-race decreases in pCO, HCO^−^, and moderate elevations in lactate confirm the substantial metabolic and ventilatory stress imposed by prolonged exertion. Collectively, these findings indicate that the capacity to maintain internal biochemical equilibrium, supported by favorable body composition, represents a fundamental determinant of performance in mountain ultramarathons.

## Supplemental Information

10.7717/peerj.21308/supp-1 Supplemental Information 1 Raw data

## Abbreviations

pH: hydrogen potential
Na^+^: sodium
K^+^: potassium
Ca^2+^: calcium
HCO_3_^−^: bicarbonate
pCO_2_: partial pressure of carbon dioxide
pO_2_: partial pressure of oxygen
Hct: hematocrit
Lac: lactate

## Additional Information and Declarations

### Data Availability

The following information was supplied regarding data availability: The raw data is available in the Supplemental File.
